# Mental health and psychiatry in Japan

**DOI:** 10.1192/bji.2024.24

**Published:** 2024-11

**Authors:** Kanna Sugiura, Yuhei Yamada, Naoyuki Kirihara, Tsuyoshi Akiyama

**Affiliations:** 1 Ushioda Clinic, Tsurumi Ward, Yokohama, Kanagawa, Japan, Email: kannasugiura@googlemail.com; 2 Porque, the Organization of Persons with Psychosocial Disabilities, Tokyo, Japan; 3 Japan National Group of Mentally Disabled People, Tokyo, Japan; 4 NTT Medical Center Tokyo, Tokyo, Japan

**Keywords:** Mental health services, Japan, psychiatric involuntary admission, integrated medical care and social support, patients and service users

## Abstract

Mental health services in Japan are fully integrated into the broader healthcare system, and patients have access to a wide range of services and specialists. In addition, psychiatry services are part of comprehensive mental healthcare that provides social support to address the whole person's needs. However, there are still challenges in the mental health system in Japan, including overuse of hospital beds and coercive practices such as involuntary admission, restraint and seclusion. This article will explore the current state of mental health services, the challenges the country still faces and the efforts being made to address these challenges.

## Overview of mental health services in Japan

Japan developed its first Mental Health Law in 1900; since then, there have been four significant reforms and minor changes. In the beginning, the law emphasised public safety and was coercive. Now, it more emphasises care and support for people with psychosocial disabilities. The law initially required people with mental health conditions to be detained at home, where it was the duty of family members to take care of them. In 1950, it became illegal to detain people with mental health conditions at home, and in the 1960s more psychiatric hospitals were built, and involuntary psychiatric admissions were encouraged. In 1988, the Mental Health Law introduced voluntary psychiatric admission, the Mental Health Tribunal and open wards. In addition, laws and services require families to act as responsible carers. In 1988, their duty to prevent suicides was removed from the Mental Health Law; in 2014, their duty to protect assets and continue medical treatments was removed; and in 2023, their duty to decide on hospital admission was removed. Services and statistics use diagnoses by doctors based on the ICD-10.

Institutionalisation is a characteristics of mental health services in Japan. Since the late 1950s, the number of psychiatric beds has grown, as policy aimed to have one psychiatric hospital in every prefecture; at maximum, there were 357 000 beds nationally in 2001. Currently, the number of psychiatric beds is 324 000, and the average hospital stay is 177 days. Of the patients occupying these beds, 38.2% cannot leave as they need medical treatment, and 41.1% cannot leave owing to financial or housing reasons.^1^ In 2004, mental health policy aimed to achieve deinstitutionalisation by further developing community-based mental health services, encouraging hospital discharge and reducing numbers of beds. National health insurance, which is reviewed every 2 years, and medical plans, which determine the function and coordination of medical services, including number of beds, continue to promote this direction, and deinstitutionalisation is moving forward slowly but surely.^2^

Psychiatry services in Japan are accessible to all citizens through national health insurance that covers mental and physical health services. This means that psychiatry services are available to everyone regardless of their financial situation; this helps to reduce disparities in access to care.^3^ More psychiatric out-patient clinics have opened, and these out-patient services are currently used by about 5 861 000 people, including 1 693 000 people with mood disorders, 1 237 000 people with neurotic disorders, 737 000 people with schizophrenia and 929 000 people with dementia.^4^ A recent change in the national health insurance reimbursement scheme provides more support for younger patients, 24 h medical services, linkage with psychologists, early detection and online services.

In 2017, the policy ‘Nimohokatsu’ (Comprehensive Regional Care System for Mental Health) was expanded to structure mental health services around integrated medical care and social support.^5^ This policy aims to provide a coordinated and comprehensive approach to healthcare and social support that incorporates physical and mental health services to enable individuals to live comfortably and be themselves, regardless of the presence or severity of mental health symptoms. It includes medical care (e.g. home nursing services), disability welfare and care (e.g. in-home care services, housework assistance), housing, social participation (e.g. employment), community mutual aid and public awareness (such as education).^6^ The government has established community mental health centres and community-based day care centres and provided home-visit services to expand access to mental health services in local communities.^7^

## Challenges and efforts in mental health services in Japan

Despite developing community-based services and introducing regulation committees, Japan still uses many hospital beds,^8^ and half of the admissions are involuntary; moreover, for people who are under involuntary admission, seclusion and restraint are common.^9^ If current law and policy continue, the government projects that the number of beds will be 238 000 by 2029. The Convention on the Rights of Persons with Disabilities (CRPD) reviewed the situation and handed its summary of findings to the Japanese government in September 2022,^10^ recommending inclusiveness, non-discrimination, participation in society and protection from abuse for people with disabilities, along with abolishing coercive practices. The Japanese government has not responded directly to it. Still, several laws have been revised and carry caveats mentioning the need for services to consider the CRPD, including a law regulating involuntary admission.^11^

Another major challenge is responding to the ageing population. Currently, 29.1% of 125.7 million people in Japan are older than 65 years, and this percentage could reach 38.4% in 2065. The entire health and social support system has to respond accordingly, including for people with dementia and delirium. In 2015, the Comprehensive Strategy to Accelerate Dementia Measures (New Orange Plan) was introduced. It is cross-governmental and encourages older individuals to stay healthy and provides support in the community according to their needs to reduce dependence on hospitals and facilities.^12^

There have been calls for greater patient autonomy and patient involvement in treatment decisions, as well as for the development of community-based mental healthcare services.^9^ Since around 2020, the perspectives of patients have also been incorporated into mental health policy development. By listening to the voices of those with lived experience of mental health issues, the government has emphasised recovery-oriented care and empowerment of patients.^6^ We are starting to see patients participating in the development of policies and services by central and local governments. In addition to these initiatives, mental health professionals and patients in Japan are developing various innovative programmes and approaches. For example, peer support programmes provide people with a safe and supportive environment to share their experiences and receive support from others with similar experiences. Furthermore, other initiatives, such as art therapy and mindfulness-based interventions, are being explored as potential alternatives to traditional medical treatments.

Further implementation of the Nimohokatsu policy through cooperation among mental health professionals, patients and policy makers could create a more supportive and effective mental healthcare system for everyone in Japan. In particular, this could involve a broader range of community-based supports, restructuring of medical and social welfare expenditure, integration of psychiatry into general medical care and specification of a roadmap towards deinstitutionalisation.
